# Clinical application of whole-genome array CGH during prenatal diagnosis: Study of 25 selected pregnancies with abnormal ultrasound findings or apparently balanced structural aberrations

**DOI:** 10.1186/1755-8166-3-24

**Published:** 2010-11-26

**Authors:** Paola Evangelidou, Carolina Sismani, Marios Ioannides, Christodoulos Christodoulou, George Koumbaris, Ioannis Kallikas, Ioannis Georgiou, Voula Velissariou, Philippos C Patsalis

**Affiliations:** 1 Department of Cytogenetics and Genomics, The Cyprus Institute of Neurology and Genetics, Nicosia, Cyprus; 2 Ultrasound and Fetal Medicine Centre, Nicosia, Cyprus; 3 Medical School, University of Ioannina, 45110 Ioannina, Greece; 4 Mitera Maternity, Gynecological, and Children's Hospital, Department of Genetics and Molecular Biology, Athens, Greece

## Abstract

**Background:**

The purpose of the study was the application and evaluation of array Comparative Genomic Hybridization (array CGH) in selected cases during prenatal diagnosis. Array CGH was applied in 25 fetal samples out of which 15 had normal karyotypes and abnormal ultrasound findings and 10 had apparently balanced structural aberrations with or without abnormal ultrasound findings. DNA was extracted from peripheral blood, chorionic villi samples (CV) and amniotic fluid. Bacterial Artificial Chromosome (BAC) array CGH (Cytochip, BlueGnome Ltd.) of 1 Mb was applied and results were confirmed with either Fluorescence In Situ Hybridization (FISH), Multiplex Ligation-dependant Probe Amplification (MLPA) or Real-Time PCR.

**Results:**

Three out of 25 samples (12%), referred for prenatal array CGH, were found to carry copy number alterations. The number of cases with clinically significant alterations was 2/25 (8%), while one (4%) was of uncertain clinical significance. Two benign Copy Number Variations (CNVs) were also found in 1/25 cases (4%).

**Conclusions:**

The outcome of this study indicates the ability of array CGH to identify chromosomal abnormalities which cannot be detected during routine prenatal cytogenetic analysis, therefore increasing the overall detection rate.

## Background

During the last 30 years conventional cytogenetics using the G-banded karyotype has been the method of choice for prenatal diagnosis, accurately detecting chromosomal abnormalities larger than 5 Mb. However, it is inefficient in detecting sub-microscopic deletions and duplications that are often associated with malformations and mental retardation. Such subtle abnormalities which cannot be detected with the conventional G-banded karyotype can be investigated and identified by array-based Comparative Genomic Hybridization (array CGH) [1,2].

Array CGH is established as the method of choice for fast and accurate detection of unbalanced structural and numerical chromosomal abnormalities [2,3]. During the last few years, the implementation of array CGH in postnatal diagnosis has been very extensive and efficient. Many reports have demonstrated the sensitivity, specificity and accuracy of this methodology detecting large and small-size imbalances [4-7]. Array CGH in postnatal diagnosis allows accurate diagnosis, characterization of syndromes, phenotype and genotype correlation, prevention, prognosis and better clinical management. The detection rate of array CGH in postnatal diagnosis was estimated between 7-11% in patients with mental retardation/Multiple Congenital Abnormalities (MCA) and with normal or no karyotype [4-7].

On the other hand, array CGH has a limited use in prenatal diagnosis. This is mainly due to the fact that CNVs detected in patients with uncharacterized genetic syndromes cannot be clearly classified as benign or pathogenic. Even though the high resolution of analysis allows the identification of smaller genetic imbalances the probability of identifying a benign variant is increased, thus imposing challenges for the interpretation of results [4]. The few studies that have applied targeted array CGH in prenatal diagnosis provide limited information as to where and under what conditions such screening should be used. These microarray platforms (Baylor College of Medicine Chromosomal Microarrays Versions 4.0, 5.0 and 6.0) targeted genomic disorders, subtelomeric as well as pericentromeric regions. Rickman et al. [8] used a targeted array CGH platform to analyze DNA samples extracted from amniotic fluid (AF) and chorionic villi (CV) cultures in pregnancies with known cytogenetic abnormalities and confirmed 29 out of 30 cases. Sahoo et al. 2006 [9] carried out array CGH in 98 prenatal samples simultaneously with G banded karyotype and detected clinically significant copy number changes in 5.1% of the samples. All five abnormalities (four cases with trisomy 21 and one case with der(7)t(3;7)) were diagnosed with both karyotype and array CGH methodologies. In the same study two additional *de novo *abnormalities (2.4%) were detected involving CNV regions of uncertain clinical significance [9]. In another study, 151 prenatal cases with normal karyotype were retrospectively screened and two causative rearrangements were identified, resulting in a diagnostic yield of 1.3% [5]. The frequencies of apparently benign alterations and findings of unclear significance were 7.9% and 0.6% respectively, after parental analysis [5]. Recently, in another study where targeted array CGH was applied in the evaluation of 300 prenatal samples, it was reported that 58 CNVs were detected. Of those, 15 (5%) were clinically significant chromosome alterations, 3 (1%) were of uncertain clinical significance, and 40 (13.3%) were benign CNVs [10]. Vialard et al. 2009 used targeted array CGH to screen for classic microdeletion syndromes and subtelomeric rearrangements in 39 fetuses with MCA after termination of pregnancy. Of those 39 fetuses, 37 had a normal karyotype and two had a *de novo *unbalanced karyotype that could not be characterized with conventional cytogenetic methods. As a result from this study, two *de novo *unbalanced karyotypes were characterized by array CGH and four additional abnormalities were diagnosed. In fetuses with normal karyotypes but MCAs the detection rate was 10.8% (4/37) [11].

Valduga et al. 2010 screened 50 fetuses with multiple malformations and a normal karyotype with 44 K oligonucleotide array (Agilent Technologies) and identified causative imbalances in five out of 50 fetuses (10%). In the same study a new polymorphic region was also found in one out of 50 fetuses (2%) [12].

Coppinger et al. 2009 [13] used either whole-genome Bacterial Artificial Chromosome (BAC) and oligonucleotide microarrays or targeted BAC microarrays on 182 and 62 prenatal cases respectively without previously known chromosome abnormalities or family history of a parent with a known chromosome rearrangement. Array analysis identified clinically significant findings in five out of 182 cases (2.7%) and a finding of unclear significance in one case (0.5%). In addition, 16 cases (8.8%) were found to have benign copy number variants. In the same study targeted array CGH analysis demonstrated detection rates of 0.9% for clinically significant abnormalities, 0.5% for findings of unclear significance and 8.0% for benign CNVs.

In this study, we present our experience of using whole-genome 1 Mb BAC array CGH during prenatal diagnosis in selected cases with abnormal ultrasound findings or an apparently balanced structural aberration and provide a summary of our results. We attempt to evaluate the role of whole-genome BAC-based array CGH in prenatal diagnosis to gain a better understanding of its clinical utility.

## Materials and methods

### Patients and Samples

All samples included in this study were received between January 2007 and November 2009 for prenatal diagnosis using G-banded karyotype and whole-genome array CGH methodology. Among the 1305 prenatal samples received within the above period only 25 cases were specifically referred by the physicians for array CGH prenatal diagnosis. Of those 25 cases, 15 had normal karyotypes and abnormal ultrasound findings and 10 had an apparently balanced structural aberration, with or without abnormal ultrasound findings (Table 1). All prospective parents were offered genetic counseling by the referring clinician and consented prior to the testing. Of the 25 fetal samples 9 were Chorionic Villi (CV) and 16 were amniotic fluid. Blood samples for DNA extraction and subsequent testing were also received from both future parents.

**Table 1:** Prenatal microarray CGH case overview

Sample Type (N = 25), CV: 9, Amniotic Fluid: 16							
**Case No**.	Reason for Referral for aCGH	U/S Findings	Sample Type	Karyotype	aCGH Result	**Inh**.	Array Result/Pregnancy Outcome
1	U/S abnormalities	Increased NT	CV	46,XY	Normal	N/A	Normal constitution. Healthy baby.

2	U/S abnormalities	Hypoplastic Nasal Bone	AF	46,XY	Dup, Dup, Del	Maternal	Two familial dups on chromosome 9 of 0.3 Mb and 0.15 Mb of unclear significance but considered to represent polymorphisms. A deletion on chromosome 17 of 1.1 Mb at the PMP22 gene consistent with HNPP. Healthy baby.

3	Familial balanced rearrangement	Normal	CV	46,XX,inv(3)(p11.2q11.2)pat	Normal	Paternal	Normal constitution. Healthy baby.

4	Familial balanced rearrangement; U/S abnormalities	Hydronephrosis, Aortic arch	AF	46,XX,inv(20)(q13.1q13.3)pat	Normal	Paternal	Normal constitution. Healthy baby.

5	Familial balanced rearrangement	Normal	CV	46,XY,inv(2)(p11.2q34)mat	Normal	Maternal	Normal constitution. Healthy baby.

6	*De novo *balanced rearrangement; U/S abnormalities	Echogenic heart; Clinodactyly	AF	46,XY,t(5;16)(q33;q24)dn	Normal	*De novo*	Normal constitution. Healthy baby.

7	*De novo *balanced rearrangement	Normal	AF	46,XX,t(2;12)(q31;q13)dn	Normal	*De novo*	Normal constitution. Healthy baby.

8	*De novo *balanced rearrangement; U/S abnormalities	Increased NT	AF	46,XX,t(3;14)(p13;q11.2)dn	Normal	*De novo*	Normal constitution. Healthy baby.

9	*De novo *balanced rearrangement; U/S abnormalities	Increased NT	AF	46,XY,t(17;21)(p11.2;q22.3)dn	Normal	*De novo*	Normal constitution. Unknown pregnancy outcome.

10	*De novo *balanced rearrangement	Normal	CV	46,XY,t(1;2)(q25;q21)dn	Del	*De novo*	*De novo *deletion on chromosome 1 of 0.2-1.35 Mb of uncertain clinical significance Elective termination of pregnancy.

11	*De novo *balanced rearrangement	Normal	AF	46,XY,t(3;8) (p13;q24.22)dn	Normal	*De novo*	Normal constitution. Healthy baby.

12	*De novo *balanced rearrangement; U/S abnormalities	Short Femur	AF	46,XX,t(11;13)(p10;q10)dn	Normal	*De novo*	Normal constitution. Unknown pregnancy outcome

13	U/S abnormalities	Myocardiopathy	CV	46,XY	Normal	N/A	Normal constitution Elective termination of pregnancy.

14	U/S abnormalities	Nasal Bone hypoplasia; Cardiac Anomalies	AF	46,XY	Normal	N/A	Normal constitution. Healthy baby.

15	U/S abnormalities	Tumor on left ear	AF	46,XY	Normal	N/A	Normal constitution. Healthy baby.

16	U/S abnormalities	Bilateral Hydronephrosis; Pyelic right kidney	AF	46,XY	Normal	N/A	Normal constitution. Healthy baby.

17	U/S abnormalities	Nasal bone hypoplasia; short limbs; echogenic bowel; FGR	AF	46,XY	Normal	N/A	Normal constitution. Healthy baby.

18	U/S abnormalities	Short limbs	AF	46,XY	Normal	N/A	Normal constitution Elective termination of pregnancy.

19	U/S abnormalities	Increased NT; talipes	CV	46,XY	Normal	N/A	Normal constitution. Healthy baby.

20	U/S abnormalities	Spine deformities; talipes; short femur	CV	46,XY	Normal	N/A	Normal constitution Elective termination of pregnancy.

21	U/S abnormalities	FGR; Single umbilical artery; Pyelic cyst	CV	46,XX	Normal	N/A	Normal constitution. Healthy baby.

22	U/S abnormalities	Increased NT	CV	46,XX	Normal	N/A	Normal constitution. Healthy baby.

23	U/S abnormalities	FGR	AF	46,XX	Normal	N/A	Normal constitution. Healthy baby.

24	U/S abnormalities	Facial Cleft; Fetal abnormality	AF	46,XX	Normal	N/A	Normal constitution Premature delivery at 29 weeks due to preeclampsia. No follow up possible at the moment.

25	U/S abnormalities	Increased NT	AF	46,XX	Dup	Parents not available	Dup of 0.7 Mb found on chromosome 22, includes the Velocardiofacial/DiGeorge Syndrome region, consistent with the 22q11.2 microduplication syndrome. Pregnancy complications resulted in fetal death.

CV, Chorionic Villus Sample; AF, Amniotic Fluid Sample; U/S, ultrasound abnormalities; FGR, Fetal growth retardation; aCGH, array Comparative Genomic Hybridization; dup, duplication; del, deletion; NT, Nuchal Thickening; Inh., Inheritance; N/A, Not Applicable

### G-banded karyotype and array CGH

All samples (CV, amniotic fluid and blood) were cultured and G-banded for karyotyping using standard cytogenetic methodologies. Fluorescence In Situ Hybridization (FISH) was performed using commercially available probes according to the manufacturer's protocol (VYSIS, Co, Downers Grove, IL, USA). DNA was extracted from CV and amniotic fluid cultured cells as well as from uncultured blood using the Qiagen Mini and Blood Midi Kit respectively (Qiagen, Valencia, CA, USA) and concentration was measured using the NanoDrop spectrophotometer (NanoDrop Technologies, Inc., USA).

For array CGH, the test and reference DNA of the same gender were co-hybridized to the Cytochip (BlueGnome, Ltd., UK,) whole-genome BAC array, as previously described [14]. The Cytochip BlueGnome array is a commercially available whole-genome BAC array with a median resolution of 0.5-1 Mb (Cytochip, BlueGnome Ltd., UK, Version 2.0). The reference DNAs were derived from pooled peripheral blood leukocytes of phenotypically normal males and females (Promega, Madison, WI, USA).

The array has been designed to provide redundancy with high sensitivity and specificity for the detection of clinically significant genomic imbalances. DNA labeling and hybridization were performed as previously described [14]. The arrays were scanned with Agilent G2565B scanner and image files were quantified using Agilent's Feature extraction software (V9.5.3.1).

### Array Data and Confirmatory Analysis

Array data were analyzed using Bluefuse software analysis (BlueGnome Ltd., UK). All detected copy number changes were compared to known aberrations listed in publically available databases, such as ENSEMBL (Ensembl: http://www.ensembl.org), DECIPHER (http://decipher.sanger.ac.uk) and the Database of Genomic Variants (DGV, http://projects.tcag.ca/variation/) using NCBI136/hg18 UCSC assembly. Parental samples were analyzed by array CGH, where needed, to specifically exclude the presence or absence of CNVs detected in the fetus. For all copy number variations found, confirmatory FISH, Multiplex Ligation-dependant Probe Amplification (MLPA) or Real Time Polymerase Chain Reaction (RT-PCR) were performed using previously described standard procedures [15-17].

## Results

### Array GCH analysis and Data Interpretation

Out of the 25 cases, three (cases 2, 10 and 25), were found to have genomic imbalances (12%) (Table 1). Two were clinically significant (2/25-8%) and one was of uncertain clinical significance (1/25-4%). In addition, in one of the cases (1/25-4%) in which a clinically significant imbalance was detected, two benign CNVs were also found (case 2).

**Figure 1:**
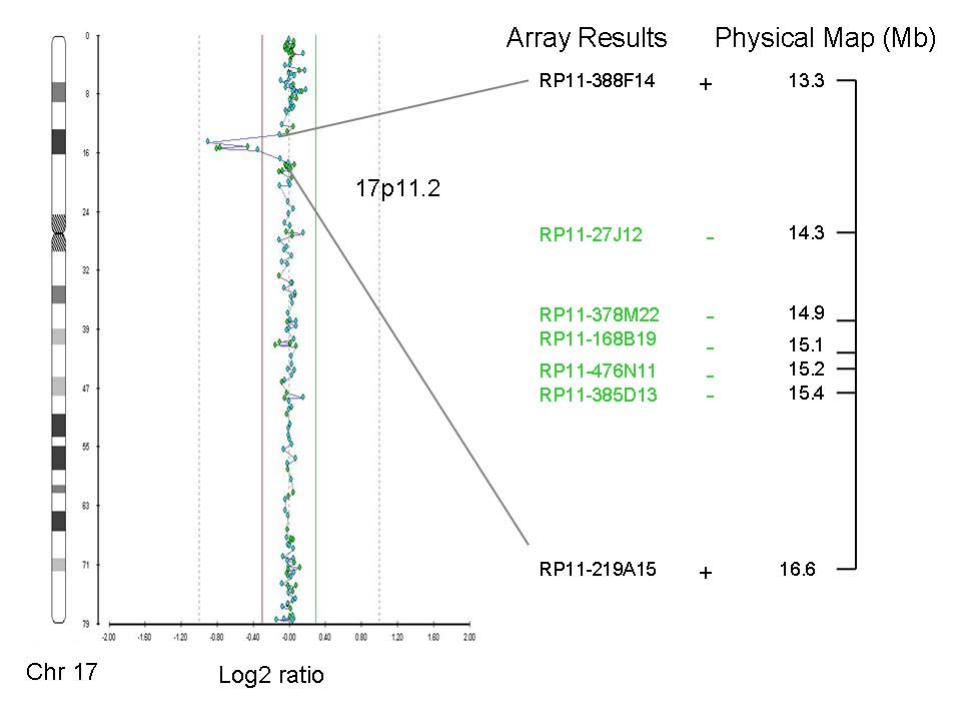
**Array CGH (Cytochip BAC array) showing a deletion on the short arm of chromosome 17 (17p11.2)**. Lines show the deleted clones and their physical location. The + or - signs indicate the presence or absence of the clone stated.

Case 2 was initially referred for prenatal cytogenetic diagnosis because of advanced maternal age. The fetal karyotype was normal but as abnormal ultrasound findings were present in a subsequent screening, it was then referred for array CGH testing. During counseling the mother revealed that she suffered from an undiagnosed mild polyneuropathy, which included episodes of numbness and weakness. Two duplications of 0.3 Mb and 0.15 Mb, and a deletion of 1.1 Mb in size were detected (Figure 1). Array CGH analysis in the parents showed that all three findings were of maternal origin. The 0.3 Mb and 0.15 Mb duplications were located on chromosome 9 between 6362145-6650803bp and 2589860-2728609bp, respectively. They were not consistent with the mother's phenotype and probably represent non-causative or benign CNVs. However, the 1.1 Mb deletion was found at the PMP22 gene located on 14324518-15415748bp on chromosome 17 (17p11.2) and is consistent with Hereditary Neuropathy with Liability to Pressure Palsies (HNPP) which is a polyneuropathy with or without symptoms, thus confirming the clinical findings in the mother who did not present any other clinical features besides the ones described for HNPP. The deletion was confirmed with MLPA in both the fetus and the affected parent. After counseling the parents decided to continue the pregnancy and they gave birth to a healthy baby boy.

**Figure 2:**
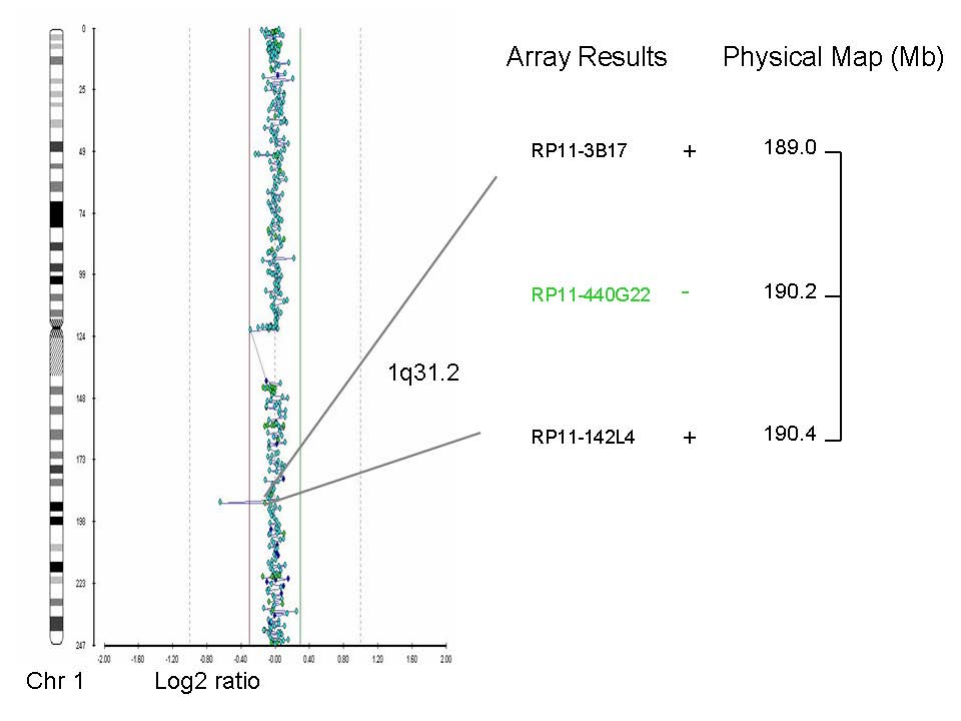
**Array CGH (Cytochip BAC array) showing a deletion on the long arm of chromosome 1 (1q31.2)**. Line shows the deleted clone as well as the previous and next non-deleted clones and their physical location. The + or - signs indicate the presence or absence of the clone stated.

Case 10 was originally referred for prenatal cytogenetic diagnosis because of advanced maternal age. An apparently balanced translocation was found during cytogenetic analysis. Parental chromosomal analysis revealed normal karyotypes and therefore the balanced translocation was classified as *de novo. *Array CGH was performed to exclude any imbalances derived from the translocation. A deletion of 0.2-1.35 Mb corresponding to clone RP11-440G22 located on 190230845bp on chromosome 1 (1q31.2) was detected at the translocation breakpoint (Figure 2). Both parents were analyzed with the same array CGH platform and were negative for the above deletion. Array-CGH results for patient and parents were confirmed using RT-PCR. The deletion was not listed in the ENSEMBL database (http://www.ensembl.org) and its clinical significance is unclear. The deleted region does not contain any genes causing known syndromes, however based on the fact that the deletion was *de novo *in origin, was located in the region of the translocation breakpoint and that there is an entry with a similar aberration in the DECIPHER database, we concluded that the deletion is probably of clinical significance and there was an increased risk for a phenotypic effect of the fetus. However, as there is no definitive evidence for the causality of this aberration, we categorized it as an abnormality of uncertain clinical significance. Pregnancy outcome was elective termination.

**Figure 3:**
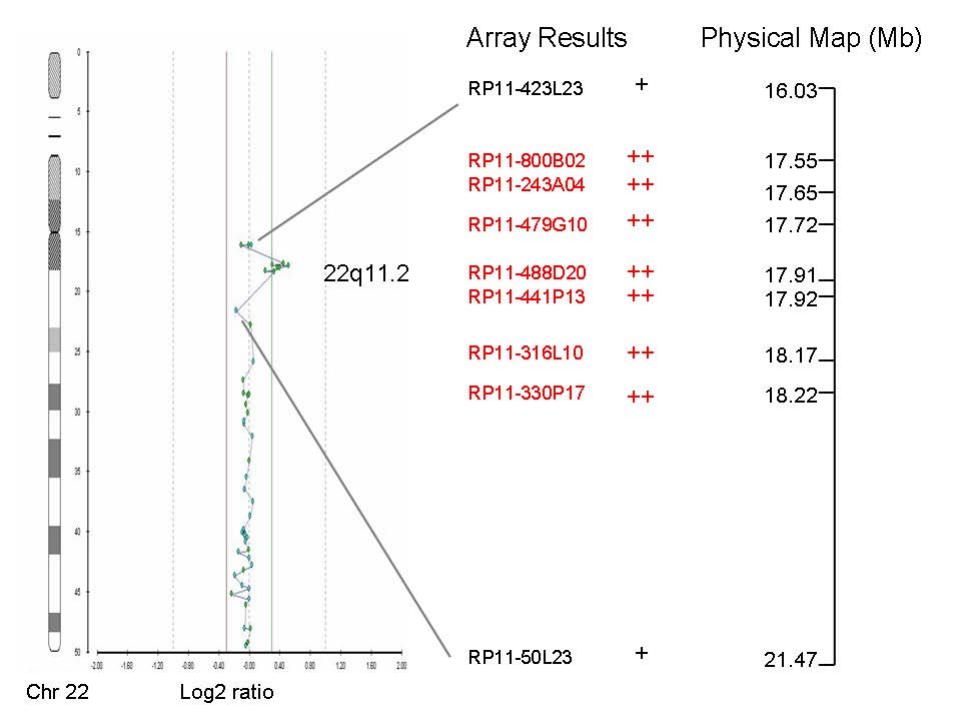
**Array CGH (Cytochip BAC array) showing a duplication on the long arm of chromosome 22 (22q11.2)**. Lines show the duplicated clones as well as the previous and next non-duplicated clones and their physical location. The + shows the presence of the clone and the ++ sign indicates duplication of the clone.

Case 25 was initially referred for chromosomal analysis due to advanced maternal age and increased Nuchal Translucency on ultrasound. When the karyotype was found to be normal it was referred for array CGH analysis. A 0.7 Mb duplication was detected which is located on 17552768-18223647bp of chromosome 22 (Figure 3). The duplication is of clinical significance because it falls within the Velocardiofacial/DiGeorge syndrome region and is consistent with the 22q11.2 microduplication syndrome [18]. The syndrome has a variable phenotype and the causative duplication can be either familial or *de novo*. The inheritance of this duplication could not be evaluated because parental screening was not performed. There were pregnancy complications which resulted in fetal death and the parents did not wish to continue the investigation.

## Discussion

This study evaluates the clinical application of whole-genome array CGH in 25 selected samples during prenatal diagnosis. Array CGH is the most advanced method yet for assessing genomic imbalances associated with genetic diseases. The implementation of genome-wide array CGH in postnatal diagnosis is the method of choice providing higher diagnostic yields and many other major advantages. However, array CGH in prenatal diagnosis is of limited use. This is mainly because trisomies and other large abnormalities (>5 Mb) are most commonly found and can easily be detected by conventional karyotyping and FISH. Furthermore, the clinical significance of many small size (<5 Mb) abnormalities usually found by array CGH may be unclear because there are syndromes caused by small size segmental imbalances that have not yet been characterized and reported. In addition, amongst the imbalances that are detected, there is a significant number of CNVs that have not yet been characterized and we are still not aware of their clinical significance, their function, their association with abnormal phenotypes and their implication in diagnosis [19,20]. Such uncertain findings may pose challenges for the clinical cytogenetic laboratory to accurately report and interpret the results of array CGH prenatal testing. As a consequence, the referring clinician may have difficulty in predicting the outcome, estimating recurrence risks and offering proper genetic counseling and clinical management. Furthermore, the anxiety of prospective parents increases while waiting for the results of such highly informative testing [21]. Another important limitation of prenatal array CGH is the fact that low level mosaicism can be present in prenatal screening but may remain undetected. The ability of array CGH to detect and evaluate low-level mosaicism is not yet known. The lowest mosaicism grade that has been detected by 1 Mb array-CGH analysis is as low as 8% [22].

In our study, we applied array CGH in only a limited highly selective number of cases (N = 25) that were originally referred for cytogenetic prenatal diagnosis (N = 1305) and were found normal or with an apparently balanced translocation or inversion. The first group consisted of cases which fetal medicine specialists had evaluated as high risk for chromosomal abnormalities because of abnormal ultrasound findings, while the second group consisted of cases in which an apparently balanced translocation or inversion was detected (Table 1). In this study we used BAC arrays for our screening for a number of reasons. The design of these arrays is more diagnostically oriented with high coverage in disease regions and less coverage in CNV regions and it is also a validated platform for all our diagnostic cases allowing an easy interpretation of results.

In a total of 25 prenatal samples three (12%) were found with confirmed copy number alterations. The number of cases with clinically significant alterations was 2/25 (8%), while the number of findings of uncertain clinical significance was 1/25 (4%). Even though comparison with the other five available studies should be done with caution as different parameters are used, the frequency of confirmed abnormalities is significant [5,9-12]. In previous studies the detection rate of clinically significant confirmed abnormalities ranged between 1.3-7.4%, while the detection rate of findings with unclear clinical significance was between 0.6-3.7% [5,9,10]. When evaluating the detection rate of prenatal diagnosis with array CGH one must take into consideration the design and resolution of the array used, the reason for referral, whether the G-banded karyotype was found normal and, finally, if non-pathogenic benign variants (CNVs) have been included when estimating the detection rate. Taking into account our results and those of others, we estimated that during prenatal diagnosis when array CGH is applied approximately up to 12% of small genomic imbalances with known or unclear clinical significance are expected to be identified. There is no doubt that such a detection rate is significant and adds important diagnostic information for prenatal genetic counseling and risk assessment. In the present study the detection rate of clinically significant aberrations is 8%. Although an aberration may not appear pathogenic, future studies may result in identification of novel causative imbalances. These findings are important for research and can provide valuable data for disease gene identification.

In our study, all three abnormalities identified following a specific referral for array CGH prenatal examination were small in size (0.2-1.5 Mb) and were not detected by conventional cytogenetic prenatal testing. This demonstrates that the application of array CGH during prenatal diagnosis increases resolution and improves the detection rate. In two of the cases (cases 2 and 25, Table 1), the abnormalities were clearly found to be consistent with specific genetic conditions. In case 2 the 17p11.2 deletion detected (HNPP) was not related to the ultrasound findings and can be characterized as a coincidental finding. In case 25 the 22q11.2 microduplication detected may or may not be associated with the ultrasound findings. The 22q11.2 microduplication syndrome has a variable phenotype and it is therefore difficult to make a correlation with the ultrasound findings when detected prenatally. Array CGH is increasingly used in prenatal testing; additional cases of prenatal 22q11.2 microduplication will eventually be detected and reported and may lead to an association with the ultrasound findings if any. In case 10, the abnormality was found to be a *de novo *deletion, with an entry in DECIPHER featuring a similar aberration. It was therefore concluded that there is an increased risk for a phenotypic effect of the fetus, even though the abnormality was of uncertain clinical significance. In all three cases, prenatal array CGH analysis added very important diagnostic information for prenatal genetic counseling and risk assessment. Elective termination of the pregnancy was only decided by the parents in case 10. Our data as well as other studies indicate that pregnancies with ultrasound abnormalities or *de novo *apparently balanced rearrangements are good candidates for prenatal diagnosis with array CGH because a large number of clinically significant cryptic abnormalities can be detected by combining ultrasound investigation and array CGH analysis [22-24]. The estimated risk for phenotypic abnormalities in carriers of *de novo *reciprocal translocations detected at prenatal diagnosis is 6%; by implementing array CGH and therefore excluding submicroscopic changes, a lower and more accurate phenotypic risk can be reported to the patient [25]. In familial reciprocal translocations as suggested by Sismani et al. 2008 [24] cryptic copy number changes at least at the resolution of 1 Mb, do not constitute a major cause for phenotypic abnormalities present in these patients where the carrier parent has a normal phenotype. The same study also demonstrated that a genomic imbalance was present in a familial case of reciprocal translocation where both the carrier parent and child had abnormal phenotypes. Therefore, array CGH should be carried out in cases of *de novo *balanced translocations, as well as in cases where the carrier parent is also affected.

Before we attempt evaluation of the use of whole-genome array CGH for prenatal diagnosis we should stress that pre-test education and counseling is paramount. It should be supplemented by consent documents which should include a written summary of the testing process, potential benefits and limitations, co-incidental findings, possible testing outcome and sources of educational material. Furthermore, the laboratory director, the referring clinician and the genetic counselor must be available for inquiries before and after testing [21]. These measures will facilitate analysis, resolve potential problems and limit anxiety.

## Conclusion

At a resolution of 1 Mb, the application of BAC array CGH in selected cases during prenatal diagnosis allowed the detection of 8% of known clinically significant alterations and 4% of clinically uncertain alterations, which is comparable to other published data. Our study supports that pregnancies with ultrasound abnormalities or apparently balanced translocations or inversions are very good indications for prenatal diagnosis with array CGH. Small abnormalities (0.2-1.5 Mb) were found, demonstrating that array CGH is a method of high resolution and detection rate, and adds important diagnostic information for prenatal genetic counseling and risk assessment. Currently, clinical application of array CGH as a universal routine test for prenatal diagnosis is premature. Further investigation will allow an evaluation between the overall diagnostic yield of array CGH over routine prenatal testing with conventional karyotype, as well as cost effectiveness issues.

## Competing interest Statement

The authors declare that they have no competing interests.

## Authors' contributions

PE drafted the manuscript, performed array CGH and karyotyping. CS performed array CGH, participated in the coordination and helped to draft the manuscript. MI, CC, GK performed array CGH and relevant confirmations. IK performed clinical evaluation of the pregnancies. IG participated in the coordination of the project and helped to draft the manuscript. VV performed clinical evaluations of the pregnancies as well as karyotyping. PCP conceived the study, participated in its design and coordination and also approved the manuscript. All authors have read and approved the manuscript.
